# Morbidity Patterns of Non-Traffic Unintentional Injuries Among the Pediatric Age Group Attending the Emergency Department at King Abdul-Aziz Medical City, Riyadh, Saudi Arabia

**DOI:** 10.7759/cureus.9258

**Published:** 2020-07-18

**Authors:** Mohammed A AlAteeq, Abdullah K Alsulayhim, Fahad AlHargan, Ibrahim S AlSamaani, Mohammed Alyousef, Abdulrahman AlDossari

**Affiliations:** 1 Family Medicine, Ministry of National Guard - Health Affairs, Riyadh, SAU; 2 Family Medicine, King Abdullah International Medical Research Center, Riyadh, SAU; 3 Family Medicine, King Saud Bin Abdulaziz University for Health Sciences, Riyadh, SAU

**Keywords:** pediatric, trauma, disability

## Abstract

Objectives

The aim of this study is to measure the magnitude and describe morbidity pattern, management, and outcome of non-traffic unintentional injuries among a pediatric age group at a tertiary hospital in Riyadh, Saudi Arabia.

Materials and Methods

This is a retrospective descriptive cross-sectional study conducted at Emergency Department (ED) of King Abdul-Aziz Medical City, Riyadh, Saudi Arabia, including all pediatric patients aged 0 to 14 years who had non-traffic unintentional injuries and admitted to the ED from January 1, 2016, to December 31, 2017. The number of children included for the specified period was 491 patients.

Results

A total of 491 patients were included over the study period; the majority were males (64%). The most common injury types were fractures, dislocations, and subluxations (47.3%) followed by penetration injuries (21%) and burn injuries (17.5%). The most involved body site was the upper limbs (45.2%) followed by head and neck (24.2%) and lower limbs (17.3%). Fall was the leading mechanism of injuries (47.7%) followed by hot liquids and chemical exposure (14.5%). Most of the cases resulted in no significant disabilities (40%), 21.6% resulted in short-term disability, 24.2% had long-term disability, and 12.8% had permanent disabilities. There were six cases (1.2%) of mortality.

Conclusions

Non-traffic unintentional pediatric injuries are common with significant morbidity and complications, and most of them are preventable. More efforts are needed to increase public awareness and to implement preventive measures at households and public places.

## Introduction

Non-traffic unintentional pediatric injuries, which are defined as injuries that occur under accidental circumstances, excluding motor traffic accidents, are common causes of morbidity, disability, and even mortality [1]. The injuries are often caused by falls, burns, ingestion of foreign bodies, and sport-related activities. Although injuries can happen to any child due to their natural behavior, there are some factors that might contribute to the occurrence of these injuries, such as lack of parental supervision, unsafe home environment, and lack of safety measures at home.

In a Canadian study, researchers investigated records from the Canadian Hospitals Injury Reporting and Prevention Program (CHIRPP) in Kingston, Ontario, and reviewed the child injury patterns for all cases presented from 1999 to 2002. In that study, head injuries were most often observed among infants, whereas open wounds and fractures were more frequently represented in the 60- to 83-month age group. Head/facial and lower extremity fractures were common among infants, whereas upper extremity fractures were predominantly seen in the older age groups [2].

In “Deaths: Final Data for 2002” report, which documents U.S. deaths and death rates according to demographic and medical characteristics, it was found that 23 infants die among every 100,000 due to unintentional injuries, with 90% of them occurring at home [3].

In a retrospective study, analysis of data for infants ≤ 12 months of age from the American National Electronic Surveillance System-All Injury Program for 2001-2004 was performed, which revealed that falls were the major cause of these injuries and that the most common diagnoses were from contusions followed by laceration, foreign bodies, hematoma, fractures, and puncture injuries [4].

Another study analyzed the non-accidental injuries data collected at school from the National Canadian Pediatric Trauma Registry. Among 1,558 injured children, the majority were male (10-14 years old), and the leading causes were sports activities affecting the extremities (41.3%) and head and neck (39.2%) [5]. A study in the United States analyzed the snow-sports injuries of children attending the emergency department (ED) for 14 years from 1996 to 2010 and reported the total number of injuries as 78,538, of which 77% were intracranial injuries [6].

In Saudi Arabia, a retrospective study collected data on burn injuries among the pediatric age group attending the ED at King Abdul-Aziz Medical City (KAMC), Riyadh, Saudi Arabia. The study found the annual rate of injuries to be 4.9/1,000 patients; children aged one to three years accounted for most of the burn cases (48.6%), with scaled burns being the leading cause of injuries, especially from hot water and drinks, and 35% of cases occurred at home [7].

In another study in Saudi Arabia that reviewed 361 cases of hand fractures, 46% of cases were found among the 13- to 18-year-old group, and the most frequent place of injuries for the one- to four-year-old age group was at home (81.3%). The most common causes for the 1- to 8-year-old group were door slams, falls at home for 9- to 12-year-old group, and sports and falls at home for 13- to 18-year-old group [8].

To our knowledge, few studies have been conducted, and little is known about this topic in Saudi Arabia. This study aims to estimate the magnitude and describe morbidities, management, and outcome of non-traffic unintentional injuries among the children attending the ED at KAMC.

## Materials and methods

This is a descriptive cross-sectional study conducted in the pediatric ED at KAMC.

KAMC is a tertiary hospital and advanced trauma center that serves the population of the national guard and their dependents in addition to patient referrals from all over the Kingdom. The pediatric ED at KAMC has a total of 21 certified consultants and specialists, treating around 40,000 cases per year.

The population of the study comprised children between 0 and 14 years old, both genders, attending and admitted to the pediatric ED at KAMC with unintentional and non-traffic injuries. The upper limit of the pediatric age group at KAMC is 14 years.

Data were collected for the period from January 1, 2016, to December 31, 2017. All cases attending the pediatric ED and admitted during the study period due to unintentional and non-traffic injuries were included, with no randomization.

This is a chart review study. After obtaining permission, data were collected from the electronic medical records system (BestCare) at KAMC using a predesigned data collection sheet. The sheet includes three sections: the first section for age and gender, the second section for the injury description (place and activity during injury, type, mechanism, and body part involved), and the third section for the injury management and clinical outcome.

Data were entered and analyzed using Statistical Package for the Social Sciences (SPSS) Version 20, (IBM Corp., Armonk, NY, USA). Descriptive statistics were performed in the form of frequencies and percentages for categorical variables, whereas mean and standard deviation were used for the description of continuous variables. We used a chi-square test to assess differences between categorical variables. Means were compared using an independent Student’s t-test (analysis of variance when applicable). Statistical significance is set to less than 0.05.

Approval of the study was obtained from King Abdullah International Medical Research Center. All the information of the patient was kept confidential and used only for research purposes. The data were stored in password-protected computers, and only research members had access to it.

## Results

The total number of children who attended and were admitted due to non-traffic unintentional injuries was 491. A total of 579 injuries were seen among them, with 540 having body sites affected. Of them, 56% were less than 5 years old, 28.3% were 5-10 years old, and 15.7% were more than 10 years old. Males made up the majority of patients (64%).

Table 1 represents the different types of injuries, body sites involved, type and place of activity during the injury, and mechanism of injury. Fractures, dislocations, and subluxations were the most common injuries found in all age groups (47.3%) followed by the burn in the 0-5 years age group (24.7%), and cut, laceration, and open wound in the 5-10 and >10-year-old groups (25.9% and 29.9%, respectively).

**Table 1:** Type, site, activity, place, and mechanism of non-traffic unintentional injuries among children attending the emergency department, King Abdul-Aziz Medical City, 2017

		No	%
Type of injury	Fracture/dislocation/subluxation	232	47.3
	Cut/laceration/open wound	103	21.0
	Burn	86	17.5
	Brain injury/intracranial bleeding	63	12.8
	Near drowning	20	4.1
	Others	75	15.2
	Total	579	117.9
Site of injury	Upper limbs	222	45.2
	Head and neck	119	24.2
	Lower limbs	85	17.3
	Multiple or whole body	65	13.2
	Chest	17	3.5
	Abdomen	12	2.4
	Others	20	4.1
	Total	540	109.9
Activity at time of injury	Leisure	89	18.1
	Daily activities	111	22.6
	Others	5	1.0
	Unknown	286	58.2
	Total	491	100.0
Place of injury	Home	100	20.4
	Others	23	4.7
	Play area	19	3.9
	Chalet/resort	17	3.5
	Unknown	332	67.6
	Total	491	100.0

**Figure 1:**
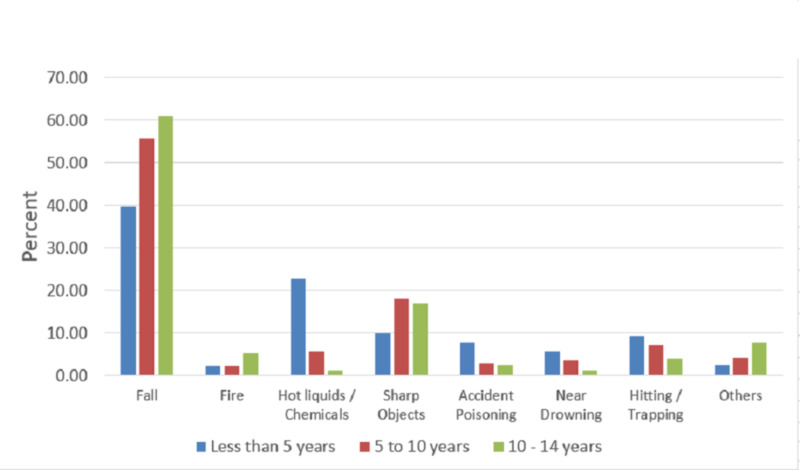
Age versus mechanism of non-traffic unintentional injuries among children attending the emergency department, King Abdul-Aziz Medical City, 2017

Upper limbs were the most injured parts of the body (45.2%) followed by head and neck (24.2%) and lower limbs (17.3%). Fall was the leading cause of injuries in all age groups followed by exposure to hot liquids and chemicals in the zero- to five-year-old group, whereas sharp objects were the second most common mechanism of injury in children above five years old (Figure 1).

In the sub-analysis of involved body site according to age, head and neck was the most common site of injury in patients from 0 to 10 years, whereas lower limbs were the most common in those above 10 years of age (Table 2).

**Table 2:** Commonest types of non-traffic unintentional injuries and body site involved among children attending the emergency department, King Abdul-Aziz Medical City, 2017

Age group	Type of injuries				Body site involved		
	Cut/laceration/open wound	Fracture/dislocation/subluxation	Burn	Brain injury/bleeding	Head and neck	Multiple or whole body	Lower limbs
Less than 5 years	44	112	68	48	80	47	36
	16.0%	40.7%	24.7%	17.5%	29.1%	17.1%	13.1%
5 to 10 years	36	75	13	9	26	12	25
	25.9%	54.0%	9.4%	6.5%	18.7%	8.6%	18.0%
Above 10 years	23	45	5	6	13	6	24
	29.9%	58.4%	6.5%	7.8%	16.9%	7.8%	31.2%
Total	103	232	86	63	119	65	85
	21.0%	47.3%	17.5%	12.8%	24.2%	13.2%	17.3%
p-Value	0.007	0.004	0.00	0.002	0.017	0.017	0.001

In most cases, the type of activity during an injury was not reported. When reported, daily activity was the most common across all age groups and in the age group below five years (24.7%), whereas other age groups got injured mostly during leisure times (22.3% and 22.1%, respectively). In most cases, the place of injury was not reported. When reported, the home was the most commonplace of injury across all age groups (Tables 1, 3).

**Table 3:** Age correlation with type and place of activity associated with non-traffic unintentional injuries among children attending the emergency department, King Abdul-Aziz Medical City, 2017

Age group	Type of activity				Place of activity				
	Leisure	Daily activities	Others	Unknown	Home	Chalet/resort	Play area	Others	Unknown
Less than 5 years	41	68	0	166	69	11	5	6	184
	14.9%	24.7%	0.0%	60.4%	25.1%	4.0%	1.8%	2.2%	66.9%
5 to 10 years	31	29	5	74	25	5	8	8	93
	22.3%	20.9%	3.6%	53.2%	18.0%	3.6%	5.8%	5.8%	66.9%
Above 10 years	17	14	0	46	6	1	6	9	55
	22.1%	18.2%	0.0%	59.7%	7.8%	1.3%	7.8%	11.7%	71.4%
Total	89	111	5	286	100	17	19	23	332
	18.1%	22.6%	1.0%	58.2%	20.4%	3.5%	3.9%	4.7%	67.6%

Most patients who attended the ED were admitted to wards, intensive care units (ICUs), or burn units (89.2%), whereas 10.6% were admitted for emergency surgery; one patient died on arrival to the ED (0.2%) (Table 4). Six cases of death were reported; one died on arrival to the ED and five after admission.

**Table 4:** Management and outcome of non-traffic unintentional injuries among children attending the ED, King Abdul-Aziz Medical City, 2017 ED, emergency department

		No	%
Management	Admitted to ward/burn unit/intensive care unit	438	89.2
	Admitted for emergency surgery	52	10.6
	Died on arrival to ED	1	0.2
	Total	491	100.0
Outcome	No significant disability	197	40.1
	Short-term disability (<6 weeks)	106	21.6
	Long-term disability (>6 weeks)	119	24.2
	Permanent disability	63	12.8
	Death	6	1.2
	Total	491	100.0

As a complication of injury, 40% of patients had no significant disability, 24.2% had long-term disability, 21.6% had short-term disability, 12.8% of injuries resulted in permanent disability, and 1.2% died because of injury (Table 4).

**Figure 2:**
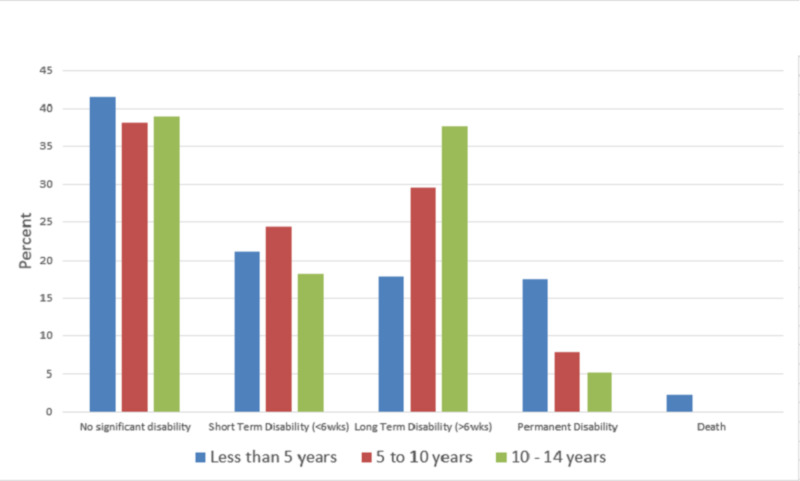
Age versus outcome of non-traffic unintentional injuries among children attending the emergency department, King Abdul-Aziz Medical City, 2017

The outcome of injuries according to different age groups is represented in Figure 2.

Tables 5 represents the management and outcome of the top four types of injuries.

**Table 5:** Management and outcome of the top four types of non-traffic unintentional injuries among children attending ED, King Abdul-Aziz Medical City, 2017 ICU, intensive care unit; ED, emergency department

Type of injury	Type of management			Outcome of injury				
	Admitted to ward/burn unit/ICU	Admitted for emergency surgery	Died in ED	No significant disability	Short-term disability (<6 weeks)	Long-term disability (>6 weeks)	Permanent disability	Death
Fracture, dislocation, subluxation	199	33	0	62	64	102	4	0
	85.7%	14.2%	0.0%	26.7%	27.6%	44.0%	1.7%	0.0%
Cut, laceration, open wound	87	16	0	45	35	17	6	0
	84.5%	15.5%	0.0%	43.7%	34.0%	16.5%	5.8%	0.0%
Burns	86	0	0	31	2	0	52	1
	100%	0.0%	0.0%	36.0%	2.3%	0.0%	60.5%	1.2%
Brain injury/bleeding	60	2%	1	34	12	9	3	5
	95.2%	3.2	1.6%	54%	19.0%	14.3%	4.8%	7.9%

## Discussion

Non-traffic unintentional injuries are common and considered a major cause of morbidity and mortality among children. This study aimed to describe the morbidity, management, and outcome of non-traffic unintentional injuries admitted to one of the largest pediatric ED in the region.

The reported rate of admission of children due to non-traffic unintentional injuries in this study was found to be 491 cases in two years, with almost five admissions every week. If this number is combined with the number of admissions due to traffic road accidents and child abuse and neglect, this represents a considerably high number that needs special attention.

Previous similar local studies addressed either specific types of injury, such as fractures or burns, or all types of injuries with no specification. For example, in a local study done by Alnasser et al., among the pediatric trauma cases attending the same center for the same age group, 50% of blunt trauma was attributable to road traffic accidents. Although not mentioned clearly, this number included all cases either admitted to a ward/ICU/emergency surgery or managed in ED and discharged home [9].

In a study conducted by Alharthy et al. in the same hospital [7], the rate of burn injuries was 4.9/1,000/year compared to this study, which reported an incidence rate of 2.15/1,000/year. The difference could be attributed to our study reported the admitted cases only.

Likewise, in another hospital in Riyadh, Saudi Arabia, the reported hand fracture cases for patients 18 years of age and below were almost 60 cases per year [8]. In our study, fractures were not reported alone but within musculoskeletal injuries that include fractures, dislocations, or subluxations, and the number of cases was 232, thus being the most common type of injury in all age groups.

Internationally, and for comparison, 77,500 cases were reported in Iran from all over the country for children below the age of seven years, of which 99.7% reported as unintentional, and 56.6% of them were boys [10]. The reported rate of unintentional injuries in Kampala, Uganda, in a tertiary hospital was 556 cases in five months, including injuries resulting from road traffic accidents [11].

Most cases in this study were boys (64%), similar to what was reported in other studies [8-10]. This is expected since boys typically participate in more outdoor activities and are involved in more risky behaviors. Similar figures were reported by Alnasser et al., Esfanjani et al., and Alnasser et al., with boys constituting 68%, 56.6%, and 74.3% of trauma cases, respectively [9-10,12].

More than half of the cases were less than five years old. This was also reported by Alharthy et al. [7], where the majority of burn cases were in children less than three years old, compared to study from Iran which reported That 70% of cases were of children below five years old [10]. This is a worrying issue since injuries in this age group of children most likely are a result of poor home safety or lack of adult supervision.

When reviewing the type of injuries in this study, one in four children aged five years or below had burn injury, which was the second most common type after musculoskeletal injuries, which was the most common across all age groups. Similar findings were found by Alharthy et al., who reported 48.6% of burn injuries occurred in children between one and three years of age [7].

Data extracted from the Hospital-Based Trauma Registry at KAMC, and reported by Alghnam et al. showed that falls were the common type of non-traffic injuries and were more common among boys [13]. Likewise, in an old local study, the most common type of injuries for children below the age of four years was fall [14]. This is in agreement with our findings, where fall was the most common mechanism of injuries.

Comparing to other regional studies, one study was conducted in AlAin, United Arab Emirates (UAE), which reported that the most common types of injury among children less than five years of age were falls (14.1%), blunt trauma (4.4%), and burns (4.2%) [15].

An interesting study was conducted in India to review domestic accidents, in which those authors performed 796 household surveys. Families reported the occurrence of accidents at home or immediate surroundings for children aged 14 years and below. This is similar to the findings of our study. The rate of domestic accidents was 2.7%, and the most commonly reported accident was fall, which was associated with younger age and female gender [16]. This is different from findings of our study, in which injuries occurred more among boys. A study using a similar methodology was conducted in Bangladesh and showed that the most common type of injury was drowning and falls. Similar to our study findings, this study found injuries higher in boys and that homes were the most common site of injury [17].

Considering the body part involved in the injury, upper limbs were the most common site of injury by fall for all ages (45%). With sub-analysis, 29.1% of injuries in children below five years of age involved the head and neck. This is again worrying since serious complications can be expected when the head and neck are injured. Alnasser et al. found that the involved body part was reported only for penetration type injury and that the most common body sites involved were the extremities followed by the face and orbit [9].

For most of the cases, the type of activity during the injury and the place at which the injury occurred were not reported (58.2 and 67.6%, respectively). This could be either due to incomplete information gathering during the evaluation process or due to lack of documentation by health care staff. This needs to be noticed and corrected during the process of documentation of cases by ED staff since it is important for the management of cases and prevention of future injuries. In the reported cases of activity during the trauma, most of the children aged five years and above were engaged in leisure activities. This is expected since children in this age group mobilize more and are exposed to more blunt injuries compared to younger kids.

When the place of injury was reported, the home was the most commonplace in all age groups. Likewise, data form Alharthy et al. shows that most burn injuries (35%) are typically sustained at the home compared with 2.7% that occurred outside the home. [7]. This implies a need for greater responsibility for parents and guardians in the prevention of childhood injuries. Home safety for children is not a well-covered issue neither by health care workers nor by other related parties in this community. More has to be done to raise public awareness and to implement trauma preventive measures at homes and public places.

It is reassuring that the vast majority of cases did not need emergency surgery, and this might reflect the low level of seriousness of injuries. Around 10% of cases were in need of emergency surgery, which was performed mainly for cases of musculoskeletal and open wound injuries. Unfortunately, none of the local studies reviewed reported the type of management performed for the reported injuries.

The majority of affected children in this study were under five years of age, and injuries occurred at home, which indicates the importance of practicing home safety for younger children; prevention efforts should be prioritized to ensure home safety for younger kids. Mack et al. suggested using the Health Impact Pyramid model to implement preventive measures for unintentional injuries [18]. In this approach, injury prevention would be enhanced by “multilevel modifications of individual’s behavior, public policies, laws and enforcement, environment, consumer products, and engineering standards” [19].

One of the limitations of this study is that it did not include the injury cases that were managed at the ED and discharged home. In addition, there was a lack of documentation for most of the cases for the place and type of activity when the injury occurred. The third limitation is the age range where the upper limit was set to be 14 years, whereas the upper limit internationally is 18 years.

## Conclusions

Non-traffic unintentional pediatric injuries are common with significant morbidity and complication, and most of them are preventable. More efforts are needed at family and community levels to increase public awareness and to implement prevention measures at households and public places.
